# A Hydrogel Culture System Regulates Human Adipocyte Function

**DOI:** 10.3390/ijms262210865

**Published:** 2025-11-09

**Authors:** Jason Junhyoung Kwon, Jie Li, Joshua Yu-Chung Liu, Christopher Patsalis, Peizi Wu, Yoshiki H. Kawase, Alexander Ky, Carter Pasternak, Damian D. Mason, Nadejda Bozadjieva-Kramer, Claudia Loebel, Carey N. Lumeng, Robert W. O’Rourke

**Affiliations:** 1 Department of Surgery, University of Michigan Medical School, Ann Arbor, MI 48109, USA; kwonju@umich.edu (J.J.K.); jieltian@umich.edu (J.L.); yokawase@umich.edu (Y.H.K.); pasterna@umich.edu (C.P.); nibozad@med.umich.edu (N.B.-K.); 2 Department of Bioengineering, University of Pennsylvania, Philadelphia, PA 19104, USA; yuchungl@seas.upenn.edu (J.Y.-C.L.); loebelcl@seas.upenn.edu (C.L.); 3 Department of Bioinformatics, University of Michigan, Ann Arbor, MI 48109, USA; 4 Department of Pediatrics and Communicable Diseases, University of Michigan Medical School, Ann Arbor, MI 48109, USA; 5 Department of Surgery, Veterans Affairs Ann Arbor Healthcare System, Ann Arbor, MI 48105, USA; 6 Graduate Program in Immunology, University of Michigan Medical School, Ann Arbor, MI 48109, USA; 7 Graduate Program in Cellular and Molecular Biology, University of Michigan Medical School, Ann Arbor, MI 48109, USA

**Keywords:** adipocyte, adipogenesis, hydrogel, extracellular matrix, lipid storage

## Abstract

Alterations in the adipose tissue extracellular matrix are well established in obesity, but the role of matrix mechanics in regulating adipocyte function is not well understood. We used a hydrogel–adipocyte culture system to study the effects of matrix stiffness on adipocyte function. We found that intermediate matrix stiffness approximating native human adipose tissue promoted maximal adipogenesis and lipid storage, while stiffness above or below this threshold impaired adipogenesis and lipid storage. Transcriptomic analysis revealed that matrix stiffness regulated diverse signaling pathways in adipocytes related to lipid metabolism, immunity and inflammation, angiogenesis, and extracellular matrix interactions. These data elucidate the role of matrix mechanics in regulating adipocyte function and will guide further studies towards developing optimal matrix characteristics for adipocytes in in vitro culture models and designing adipocyte delivery vehicles for in vivo translational applications.

## 1. Introduction

Rigorous evidence implicates alterations in the extracellular matrix (ECM) in adipose tissue dysfunction in obesity and metabolic disease, but the underlying mechanisms are poorly understood. It is postulated that increased ECM deposition leads to increased adipose tissue stiffness, which in turn impairs adipocyte function, but data supporting this hypothesis is conflicting. While some studies document increased histologic and biochemical measures of adipose tissue ECM deposition in obesity and metabolic disease [1,2,3,4,5], others demonstrate the opposite [6,7,8,9]. In addition, despite the extensive data supporting an association of subcutaneous adipose tissue (SAT) with metabolic health and visceral adipose tissue (VAT) with metabolic disease, reports demonstrate increased stiffness and ECM deposition in SAT relative to VAT in obese humans [9,10]. These conflicting data speak to a complex relationship between adipocyte function, matrix composition, and tissues’ mechanical properties, and highlight the knowledge gap regarding the precise mechanisms by which ECM regulates adipose tissue function.

In vitro culture systems are important tools for dissecting the role of matrix features in regulating cell function. Our group has previously used a native adipose tissue ECM–adipocyte culture system to demonstrate that depot- and disease-specific features of adipose tissue ECM can modify adipocyte stromal cell (ASC)/mature adipocyte function [11,12], but these systems do not permit dissection of the separate roles of ECM biochemical/protein composition from matrix mechanics in regulating cell function. Artificial matrix–cell culture systems provide a tool to address this question. Adipocyte function has been studied in artificial matrix cultures, and while data is conflicting, stiffer underlying matrix substrates tend to have detrimental effects on adipocyte differentiation and metabolic function [13,14,15,16,17]. However, most studies utilize 2D culture systems, which permit cell spreading that is independent of underlying matrix crosslinking. In contrast, in 3D environments, matrix stiffness and spatial crosslinking are coupled and directly correlated, i.e., as stiffness increases, so do the spatial constraints on cell spreading, a more accurate model of in vivo tissue.

Previous work by our group studied the role of matrix stiffness in regulating adipocyte lipid accumulation in a 3D hydrogel culture model and determined that intermediate matrix stiffness optimized adipocyte lipid storage [18]. Our goal here was to extend these investigations to study adipocyte function over a range of matrix stiffnesses, using a similar but more tractable commercially available dextran-hydrogel matrix culture system that could be widely used by multiple investigators. This hydrogel system has no cell-adhesive moieties and is enzymatically degradable via matrix metalloprotease (MMP)-sensitive crosslinkers. We hypothesized that these studies would define matrix mechanical properties that optimize adipocyte metabolic function as a first step towards developing tailored matrix vehicles for more accurate in vitro and in vivo modeling of native adipose tissue. We demonstrate that intermediate matrix stiffness is associated with maximal adipogenesis and maximal adipocyte lipid accumulation, while higher or lower matrix stiffnesses impair adipogenesis and lipid accumulation and promote a fibrotic cell phenotype. These data elucidate the complex relationship between matrix mechanics and adipocyte function.

## 2. Results

### 2.1. Rheologic Properties of TRUE7 Hydrogels Approximate Native Human Adipose Tissue

**Figure 1:**
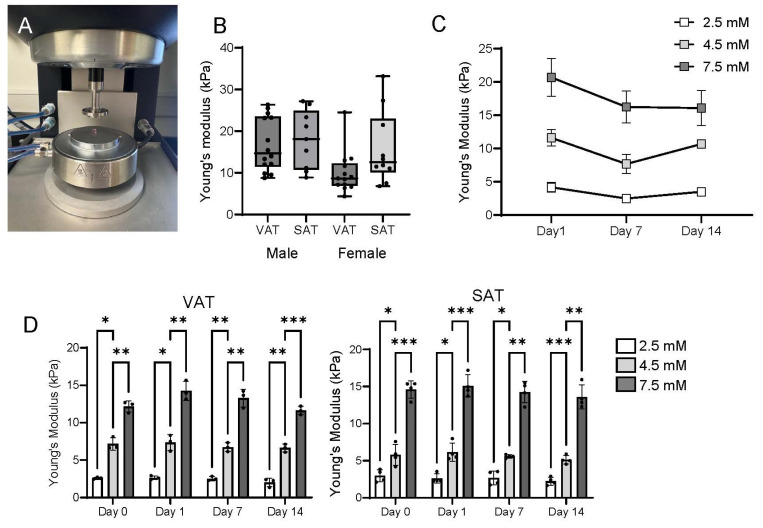
Rheological properties of TRUE7 hydrogels: (**A**) HR30 Discovery Hybrid Rheometer (TA Instruments Inc., Newcastle, DE, USA); (**B**) compression data of matched human VAT and SAT from 13 male and 12 female NDM human subjects. Boxes: 25–75 percentiles; line: median, whiskers: range. No statistically significant difference between the four groups (VAT-Male, SAT-Male, VAT-Female, and SAT-Female, ANOVA). Each data point represents the mean of 3–5 measurements of stiffness for each tissue sample from an individual patient. (**C**) Compression data from acellular TRUE7 hydrogels of 3 different RGD formulations (2.5 mM, 4.5 mM, and 7.5 mM) designed to approximate 25%, the mean, and 75% of human adipose tissue. Differences between 4.5 mM, 2.5 M, and 7.5 mM were significant (*p* < 0.05) at all timepoints. (**D**) Compression data of hydrogels seeded with VAT (bottom left) or SAT (bottom right) ASC, followed by adipogenic differentiation of 14 days, with compression testing at days indicated; * *p* < 0.05, ** *p* < 0.01, and *** *p* < 0.001; pairwise comparisons between different days for the same hydrogel concentrations not shown, but were all non-significant; data from 4 NDM male subjects.

We used static rheology compression testing to define the stiffness of human adipose tissue (Figure 1A). Young’s moduli of human adipose tissue ranged from 4 to 27 kPa (median 12 kPa for VAT, 13 kPa for SAT), with no statistically significant difference between VAT and SAT (Figure 1B). We generated TRUE7 hydrogels in a range of stiffness approximating this range of Young’s moduli and performed compression testing in acellular and cellularized hydrogels to validate these formulations. We observed a modest decrease in stiffness in acellular hydrogels over a 14-day period in culture, primarily between days 0 and 7, which is consistent with some degree of intrinsic hydrogel degradation (Figure 1C). In cellularized hydrogels, while differences between the three hydrogel concentrations were preserved at each time point, no significant changes in stiffness in any single hydrogel concentration over the culture period were observed, suggesting that adipocytes do not substantially degrade hydrogels over time (Figure 1D). Together, these data support the TRUE7 adipocyte culture system for modeling the mechanical properties of human adipose tissue.

### 2.2. Matrix Stiffness Regulates Adipogenesis and Adipocyte Lipid Storage

**Figure 2:**
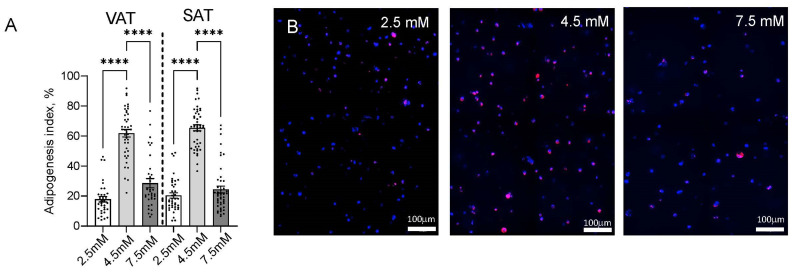
Matrix stiffness regulates adipogenesis: (**A**) ASC differentiated into adipocytes for 14 days in hydrogels, then stained with LipidTox/DAPI, imaged, and adipogenesis index calculated. Each datapoint represents % adipogenesis index for a single 20× field, calculated as # LipidTox (red)-stained cells divided by total # DAPI (blue)-stained cells; 3–6 20× fields per patient, per condition measured. Data is combined from male and female subjects; similar results are observed when stratified by sex, with no difference between sexes. **** *p* < 0.0001. A total of 12 NDM subjects, 5 male and 7 female. (**B**) Representative 20× confocal microscopy images of mature human VAT adipocytes in hydrogels from which adipogenesis indices were calculated. Red: LipidTox, Blue: DAPI.

**Figure 3:**
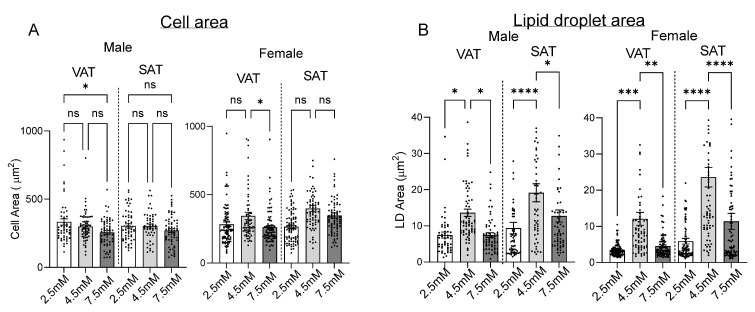

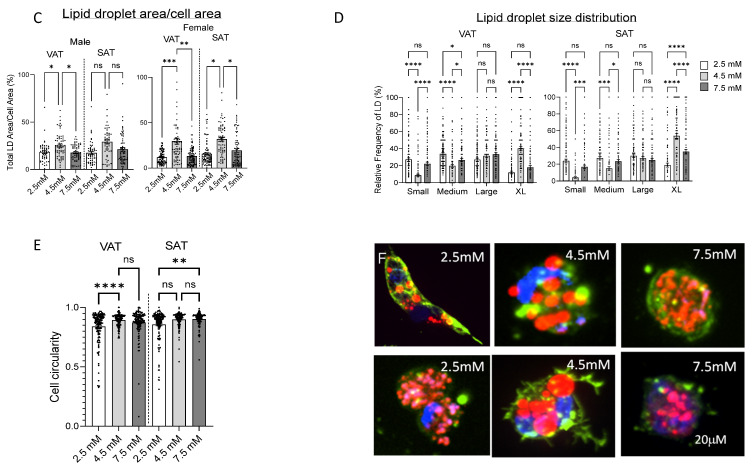
Matrix stiffness regulates adipocyte lipid storage and morphology: (**A**) Cell area (micrometer^2^ (μm^2^)) of VAT or SAT ASC seeded into hydrogels, followed by adipogenic differentiation. Each data point represents the cell area for an individual cell. (**B**) Mean lipid droplet area (μm^2^). Each data point represents the mean lipid droplet area per cell for an individual cell. (**C**) Mean lipid droplet area/cell area. Each data point represents lipid droplet area/total cell area for individual cells. (**D**) Quantification of lipid droplet (LD) size distribution in adipocytes in hydrogels using confocal microscopy, analyzed using pooled quartile-based binning. LDs were classified into four size categories based on global quartile thresholds calculated across all samples: small (<Q1), medium (Q1–Q2), large (Q2–Q3), and XL (>Q3). Quartiles were calculated using the descriptive statistics function in PRISM on the entire LD size data pool. The size ranges were small (<2.22 mm^2^), medium (2.22 mm^2^–3.59 mm^2^), large (3.60 mm^2^–8.45 mm^2^), and XL (>8.45 mm^2^). Figure shows % LDs in each size category within quantified cells for each experimental condition. Data combined from male and female subjects; similar results were observed when stratified by sex, with no difference between sexes. (**E**) Cell circularity index of adipocytes in hydrogels. Each data point represents the circularity index for an individual cell. Data combined from male and female subjects; similar results observed when stratified by sex, with no difference between sexes. (**F**) Representative confocal microscopy images of lipid droplets containing mature human VAT (top) or SAT (bottom) adipocytes in hydrogels. For 2.5 mM hydrogel images (left), top left image is representative of a smaller population of cells with low circularity, and bottom left image is representative of a majority population of cells with high circularity. Red: LipidTox, Blue: DAPI, Green: Phalloidin. ns *p* > 0.05, * *p* < 0.05, ** *p* < 0.01, *** *p* < 0.001; **** *p* < 0.0001 between indicated arms. Data from 5 male and 7 female NDM subjects.

We studied the role of matrix stiffness in regulating adipogenesis and lipid metabolism in human adipocytes using confocal microscopy with digital analysis. Adipogenesis was maximal in intermediate stiffness 4.5 mM hydrogels relative to low stiffness 2.5 mM and high stiffness 7.5 mM hydrogels (Figure 2). Adipocyte cell size was reduced in 7.5 mM hydrogels in cells from VAT but not SAT (Figure 3A). Total lipid droplet area and lipid droplet area normalized to cell area were maximal in 4.5 mM hydrogels relative to 2.5 mM and 7.5 mM hydrogels, except in SAT cells from males, in which the increase in lipid droplet area normalized to cell area in 4.5 mM hydrogels did not reach significance (Figure 3B,C). When lipid droplet size was stratified into subcategories, alterations in lipid accumulation in different hydrogel stiffnesses were primarily accounted for by decreased small lipid droplets and increased extra-large lipid droplets in 4.5 mM hydrogels relative to 2.5 mM and 7.5 mM hydrogels (Figure 3D). A small but statistically significant population of cells with decreased circularity was observed in 2.5 mM hydrogels but not in 4.5 mM or 7.5 mM hydrogels (Figure 3E,F). No significant differences were observed between male and female subjects for any lipid droplet metrics in response to hydrogel stiffness. Lipid droplet area was larger in SAT relative to VAT in the 4.5 mM and 7.5 mM hydrogel stiffness but not in the 2.5 mM hydrogel (Supplementary Figure S1). Together, these data demonstrate that intermediate hydrogel stiffness maximizes adipogenesis and adipocyte lipid storage, and that low gel stiffness induces a subpopulation of cells that are less rounded, which is consistent with greater cell spreading, possibly indicating a fibrotic phenotype, while high hydrogel stiffness reduces cell size in VAT adipocytes, which is consistent with constraints on cell hypertrophy.

### 2.3. Matrix Stiffness Regulates Adipocyte Transcriptional Programs

**Figure 4:**
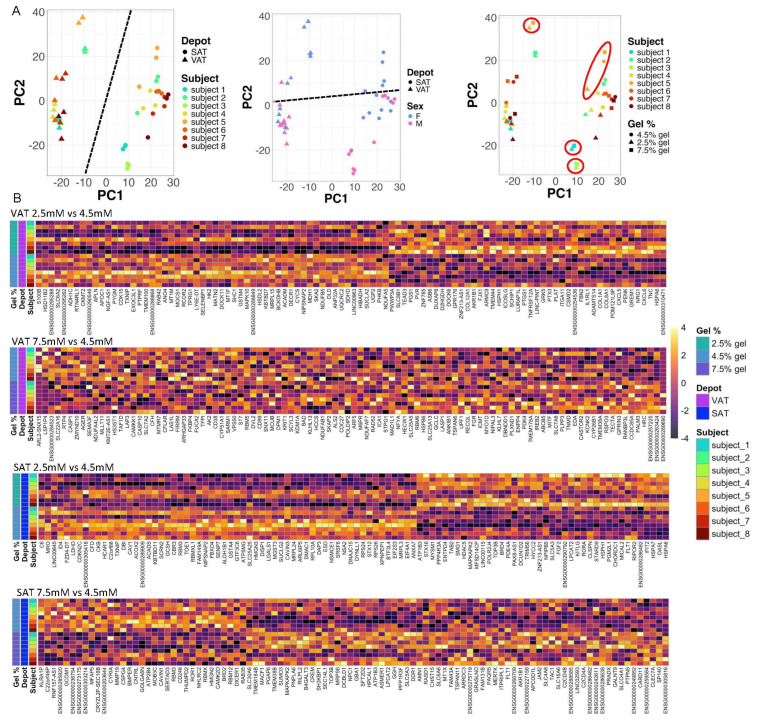
Matrix stiffness regulates adipocyte gene expression: (**A**) PCA plots of RNASeq data grouped by hydrogel % and individual patients. Dotted lines show relative clustering by depot (PC1, left) and sex (PC2, middle); circles demonstrate examples of relatively good separation of 2.5 mM hydrogels from 4.5 mM and 7.5 mM hydrogels (right). Data from 8 NDM subjects (4 male and 4 female). (**B**) Heatmaps of 100 top nominally significant DEGs (unadjusted *p*-value < 0.050) in adipocytes in hydrogels. Referent for all heatmaps is 4.5 mM hydrogel relative to either 2.5 mM or 7.5 mM hydrogels, i.e., lighter yellow indicates increased expression and darker yellow indicates decreased expression in either 2.5 mM or 7.5 mM hydrogel relative to 4.5 mM gel.

We performed an RNA sequencing (RNASeq) of VAT or SAT ASC, differentiated for 14 days into mature adipocytes from eight NDM subjects with obesity (four male and four female) in hydrogels of different stiffnesses. We compared transcriptomics results of cells from the same subjects differentiated in 2.5 mM or 7.5 mM hydrogels, each relative to 4.5 mM hydrogels. Principal component analysis (PCA) demonstrated significant clustering by subject identification number, which is consistent with significant interpatient variability. To address this, subject-ID was included as an interaction term in the DESeq2 model. PCA plots revealed relatively less robust clustering by hydrogel stiffness, with significant overlap of 4.5 mM and 7.5 mM hydrogel data, and better separation of 2.5 mM hydrogel data from 4.5 mM and 7 mM hydrogel data. Clustering by depot (VAT, SAT) was reasonable, while clustering by sex was less robust (Figure 4A). The more robust clustering between 2.5 mM hydrogel data from 4.5 mM and 7 mM hydrogel stiffness is also apparent in differentially expressed gene (DEG) heatmaps (Figure 4B). A total of 15,324, 22,216, 18,772, and 22,219 unique transcripts were identified in VAT adipocytes in 2.5 mM hydrogels, VAT adipocytes in 7.5 mM hydrogels, SAT adipocytes in 2.5 mM hydrogels, and SAT adipocytes in 7.5 mM hydrogels, respectively. Using FDR-adjusted *p*-value (*q*-value) < 0.05 to define significance, 597, 1, 2607, and two DEGs were identified in VAT adipocytes in 2.5 mM hydrogels, VAT adipocytes in 7.5 mM hydrogels, SAT adipocytes in 2.5 mM hydrogels, and SAT adipocytes in 7.5 mM hydrogels, respectively, each relative to the referent of the same matched adipocytes (VAT or SAT) in 4.5 mM hydrogels (Figure 4B, Supplementary Table S1). Restricting analysis to DEGs with log2-fold-change differential expressions < −1.0 or >+1.0 and eliminating unidentified or antisense novel transcripts, in VAT adipocytes in 2.5 mM hydrogels and SAT adipocytes in 2.5 mM hydrogels (Supplementary Table S1), the following DEGs of interest were noted: ADIRF, which encodes adipogenesis regulatory factor, a protein that promotes adipogenesis; PLIN1, which encodes the perlipin-1 protein, involved in lipid droplet formation; and AZGP1, encoding Zinc-alpha-2-glycoprotein, which stimulates lipolysis; they were decreased in expression in both VAT and SAT adipocytes in 2.5 mM relative to 4.5 mM hydrogels. The ECM-related genes that were increased in expression in 2.5 mM hydrogels included COL8A1, GREM1, PDGFRL, SPP1, and TNC in VAT adipocytes and MMP1 and SPP1 in SAT adipocytes. Expression of several genes with overlapping functions related to inflammation and angiogenesis was increased in 2.5 mM relative to 4.5 mM hydrogels, including CXCL5, CXCL6, GREM1, CEMIP, and TNC in VAT adipocytes and CXCL10, CXCL11, CCL26, CCL5, CTSS, IL1RL1, IL7R, MMP1, and SPP1 in SAT adipocytes.

**Figure 5:**
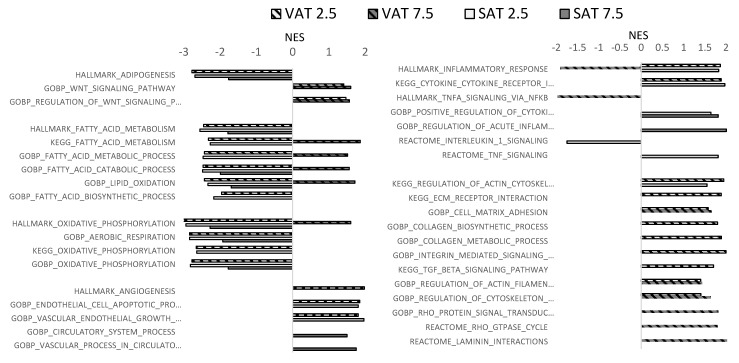
Matrix stiffness regulates adipocyte transcriptional programs: GSEA comparing 2.5 mM and 7.5 mM hydrogels to 4.5 mM hydrogel referent, i.e., (+) normalized enrichment score (NES) designates pathways enriched in 2.5 mM or 7.5 mM hydrogels and (−) NES designates pathways de-enriched in 2.5 mM or 7.5 mM hydrogels, each relative to 4.5 mM hydrogels matched for depot. Missing bar/bars for specific subgroups indicate that the pathway was not significantly regulated in that subgroup. Complete names for abbreviated pathways (designated with “…” after incomplete pathway name) can be found in Supplementary Table S2.

Next, we queried Hallmark, KEGG, Gene Ontogeny (GO)-Biological Processes, and Reactome Pathway gene sets using Gene Set Enrichment Analysis (GSEA, *q*-value < 0.25), comparing VAT or SAT adipocytes in 2.5 mM or 7.5 mM hydrogels, each relative to matched VAT or SAT adipocytes in 4.5 mM hydrogels. A total of 663, 320, 863, and 249 gene sets were significantly dysregulated in VAT adipocytes in 2.5 mM hydrogels, VAT adipocytes in 7.5 mM hydrogels, SAT adipocytes in 2.5 mM hydrogels, and SAT adipocytes in 7.5 mM hydrogels, respectively (Figure 5, Supplementary Table S2 and Figure S2). We restricted analysis to pathways in which the regulated gene size was >50, <500. Wnt-signaling pathways, which suppress adipogenesis, were enriched in all four groups (VAT and SAT adipocytes in 2.5 and 7.5 mM hydrogels relative to 4.5 mM hydrogels). The HALLMARK_ADIPOGENESIS pathway was de-enriched in VAT and SAT adipocytes in 2.5 hydrogels relative to 4.5 mM hydrogels and in SAT adipocytes in 7.5 mM hydrogels. Several fatty acid/lipid metabolism pathways were de-enriched in VAT and SAT adipocytes in 2.5 hydrogels relative to 4.5 mM hydrogels, and in SAT adipocytes in 7.5 mM hydrogels, but of note, many of these pathways were enriched in VAT adipocytes in 7.5 mM hydrogels. Similarly, pathways related to oxidative phosphorylation, aerobic respiration, and electron transport were de-enriched in VAT and SAT adipocytes in 2.5 relative to 4.5 mM hydrogels, and in SAT adipocytes in 7.5 mM hydrogels, but enriched in VAT adipocytes in 7.5 mM hydrogels. VAT adipocytes in 2.5 mM hydrogels demonstrated enrichment of several ECM/fibrosis-related pathways, including those related to collagen regulation, cell adhesion, and TGF-beta signaling; fewer of these pathways were enriched in VAT adipocytes in 7.5 mM hydrogels, although this group was enriched for Rho-GTPase signaling pathways, which plays a central role in ECM–cell interactions, and in pathways related to cell–matrix adhesion and cytoskeleton function. Fewer ECM-related pathways were regulated by SAT adipocytes, with KEGG_REGULATION_OF_ACTIN_CYTOSKELETON being enriched in SAT adipocytes in 2.5 mM hydrogels. Finally, pathways related to inflammation and, separately, angiogenesis and endothelial cell development, were enriched in VAT and SAT adipocytes in 2.5 hydrogels relative to 4.5 mM hydrogels, and in SAT adipocytes in 7.5 mM hydrogels, but de-enriched in VAT adipocytes in 7.5 mM hydrogels.

## 3. Discussion

Understanding the role of the ECM in regulating adipocyte function has important implications for generating accurate in vitro models of adipocyte culture and optimal vehicles for in vivo therapeutic cell delivery. Increasing underlying matrix stiffness in 2D culture, in most reports, promotes cell spreading and inhibits adipogenesis [14,16,17]. However, 2D culture models do not accurately model the in vivo environment, and significant differences are observed in cell responses between 2D and 3D cultures. Reports of adipocyte function in 3D cultures are sparse, with some studies showing increased adipogenesis in stiffer, highly crosslinked 3D matrices [19,20], while others show the opposite [21]. Furthermore, many reports study matrix stiffness in ranges that do not accurately model native human adipose tissue. In the present study, we measured the mechanical properties of native human adipose tissue to validate a 3D hydrogel culture system. We then used this system to determine that intermediate matrix stiffness promotes metabolically healthy adipocytes, with higher or lower matrix stiffness inhibiting adipogenesis and promoting greater cell spreading, possibly indicating a fibrotic phenotype, via matrix crosslinking regulation of cell spreading and cell shape.

Few published data describe the mechanical properties of native adipose tissue, and existing data are conflicting and use different methods. For this study, we used TRUE7 hydrogels (Sigma Inc., St Louis, MO, USA) tuned to a Young’s modulus of 0.5–20 kPa, which we validated with static rheologic compression testing. We chose this range to approximate prior estimates of native adipose tissue stiffness, including those from our group [10,22,23,24,25,26]. We also tested VAT samples from our human cohort with compression testing and confirmed stiffness in a similar range, albeit with significant variability ranging from 0.2 to 40 kPa. Nonetheless, most samples were in the range of 0.5–20 kPa (Figure 1B), consistent with published reports [10,22,23,24,25,26]. The present study was not powered to detect differences in tissue stiffness based on depot, sex, age, disease status, or other clinical characteristics, and this was not the primary goal of this report. Rather, these estimates provide a range of mechanics to which we tuned the hydrogel system, with intermediate 4.5 mM hydrogels approximating the mid-range of stiffness of human adipose tissue, while 2.5 mM and 7.5 mM hydrogels approximated the lower and upper extremes.

Our findings refute the commonly held dogma that adipogenesis increases linearly with decreasing matrix stiffness. Rather, adipogenesis and adipocyte lipid storage are maximized at intermediate matrix stiffness, with decreases in these functions at higher or lower stiffness. The prior literature on 2D culture systems supports that increased spatial constraints promote adipogenesis while decreased spatial constraints permit cell spreading, with cells adopting a more fibrotic phenotype [27]. Others have demonstrated that adipogenesis was promoted in mesenchymal stem cells in a 2D hydrogel culture, in which fibronectin concentration was titrated to limit cell spreading, an effect that was independent of hydrogel stiffness [13]. Fewer studies model 3D systems. Hogrebe et al. showed that adipogenesis in mesenchymal stem cells was inversely correlated to matrix stiffness, with a 3D environment promoting adipogenesis to a greater extent than a 2D environment at similar matrix stiffness, with cells adopting a fibrotic phenotype at matrix intermediate stiffness, and a circular phenotype at very low (0.25 kPa) and higher (10 kPa) stiffness [28]. These observations are consistent with our findings of a non-linear relationship between cell shape, adipogenesis, and matrix stiffness, but differ in that these investigators studied a lower range of matrix stiffness, likely below that of native human adipose tissue. These observations reinforce the importance of studying adipocyte function in biologically relevant 3D culture systems with mechanics similar to native adipose tissue. Our results demonstrate that matrix stiffness at the lower end of native adipose tissue stiffness (~5 kPa) is associated with lower levels of adipogenesis and more cell spreading, as evidenced by a small subpopulation of cells that demonstrated a less circular morphology at low matrix stiffness, which is similar to that observed by Hogrebe et al. at very low (0.25 kPa) and higher (10 kPa) matrix stiffnesses [28]. In contrast, adipogenesis is optimized at intermediate matrix stiffness in the range of 10–12 kPa, which we observed to be similar to native human adipose tissue, but adipogenesis was impaired at high matrix stiffness, possibly due to spatial constraints that limit cell hypertrophy. Consistent with this hypothesis, we observed smaller adipocyte size in high stiffness hydrogels in cells from VAT but not SAT, suggesting depot-specific differences in adipocyte sensitivity to spatial constraints at high matrix stiffness.

Reciprocal effects of cells on hydrogel mechanics are described [29,30,31]. Our data demonstrate a subtle decrease in the highest hydrogel stiffness over time in culture, but this was observed in acellular and cellularized hydrogels to a similar degree and did not reach statistical significance (Figure 1C,D), supporting that ASC/adipocytes do not have dramatic effects on matrix mechanics in this model system. We focused these studies of hydrogel density over time in culture (Figure 1C,D) on cells from male subjects due to limitations in human subject tissue availability. Further studies of hydrogels with differing susceptibilities to cell MMPs and other lytic enzymes and with a sex-balanced cohort will be necessary to better define the role of ASC/adipocytes in regulating hydrogel mechanics over time.

RNASeq analysis suggests depot-specific differences in transcriptional responses to matrix stiffness, including de-enrichment of transcriptional pathways promoting adipogenesis and fatty acid metabolism in low and high matrix stiffness in SAT adipocytes, and in VAT adipocytes in low stiffness matrix, which is consistent with our cell phenotype data. An exception was VAT adipocytes in high hydrogel stiffness, in which fatty acid metabolism pathways were enriched, despite the de-enrichment of adipogenesis pathways. Enrichment of fatty acid metabolism pathways in VAT at high matrix stiffness may occur in cell lineages other than adipocytes, as a shift away from adipogenesis occurs. Similarly, pathways related to oxidative phosphorylation and electron transport were de-enriched in low and high matrix stiffness in SAT adipocytes, and in low matrix stiffness in VAT adipocytes, but only one such pathway (HALLMARK_OXIDATIVE_PHOSPHORYLATION) was enriched in VAT adipocytes in high matrix stiffness, supporting qualitatively different metabolic responses to high matrix stiffness. A similar pattern was seen with inflammation-related pathways, which were enriched in low and high matrix stiffness in SAT adipocytes, and in VAT adipocytes in low stiffness matrix, but de-enriched in VAT adipocytes in high matrix stiffness. In addition, only three pathways clearly linked to inflammation were regulated in SAT adipocytes in high matrix stiffness, while multiple inflammation-related pathways were regulated in all other groups, suggesting that the inflammatory response to high matrix stiffness is attenuated in SAT relative to VAT, which is consistent with the established increased inflammatory state in VAT relative to SAT [32,33]. Fibrosis and ECM-related pathways were increased in low and high matrix stiffness in VAT adipocytes, and in low matrix stiffness in SAT adipocytes, with a greater number of ECM-associated pathways enriched in VAT compared to SAT, which is consistent with data supporting a greater fibrotic response of VAT relative to SAT [8]. Finally, pathways related to angiogenesis were enriched in VAT in low matrix stiffness and in SAT in low and high matrix stiffness, suggesting that matrix environments that are suboptimal for adipogenesis may promote differentiation down endothelial cell lineages. Our RNA sequencing analysis was designed to be hypothesis-generating, and caution must be exercised in extrapolating functional hypotheses from these data. Furthermore, PCA revealed minimal separation of data between 7.5 mM hydrogels and 4.5 mM hydrogels, along with substantial inter-subject variability, limiting study power. Further research will be necessary to confirm the specific functions of targets identified in our RNASeq dataset in regulation related to adipogenesis and lipid storage in adipocytes. Nonetheless, these data identify specific gene products and pathways as targets for future study.

## 4. Materials and Methods

### 4.1. Human Subjects

Informed consent was obtained with Institutional Review Board approval for the Veterans Affairs Ann Arbor Healthcare System under guidelines consistent with the 1964 Declaration of Helsinki and 1974 Belmont Report. VAT from the greater omentum and SAT from the abdominal wall were collected from patients with obesity (BMI ≥ 30) during bariatric surgery and processed immediately. Due to limitations in cells, cells from subsets of subjects were used for different experiments; details of subjects used for each experiment are outlined in the Figure legends. All subjects were non-diabetic (NDM), defined by no clinical history of diabetes, HbA1c ≤ 5.7%, and taking no diabetes medications per the American Diabetes Association criteria. Clinical laboratory values were measured by the clinical laboratories at the Ann Arbor Veterans Affairs Hospital (Table 1).

### 4.2. Adipose Tissue Stromal Cell (ASC) Isolation

ASC isolation and adipocyte differentiation were performed as described [34]. Briefly, adipose tissue was digested with Type II collagenase (2 mg/mL in PBS/2% BSA, Life Technologies Inc., Carlsbad, CA, USA) at 37 °C, for 60 min, and centrifuged 250 rcf, and stromal-vascular cell pellet was retrieved and plated, and adherent cells were passaged 2–3 times to enrich ASC, which were frozen in DMEM/F12, 15% fetal calf serum, and 10% DMSO in liquid nitrogen until use.

### 4.3. Hydrogel Culture

The TRUE7 system (Sigma Aldrich, St Louis MO, USA) forms a hydrogel through thioether bonds between thiol groups on a matrix metalloprotease (MMP)-cleavable crosslinker and thio-reactive groups on a dextran polymer, allowing cells to spread and migrate by degrading the network through the MMP-cleavable peptide sequence (Pro-Leu-Gly-Leu-Trp-Ala). For this study, hydrogels were formulated with the cell-degradable crosslinker (TRU-CD) at concentrations of 2.5 mM, 4.5 mM, and 7.5 mM to titrate stiffness, approximating the range of stiffness of native human adipose tissue. Hydrogels will not solidify with a crosslinker concentration of zero, so for this reason, a matrix condition with 0 mM crosslinker was not studied. For differentiation of ASC into mature adipocytes within hydrogels, 50,000 ASC were seeded in 50 µL gel and cultured in 2 mL of differentiation media (DMEM:F12 + 10 mg/L transferrin, 33 µM biotin, 0.5 µM human insulin, 17 µM pantothenate, 100 nM dexamethasone, 2 nM 3,3′,5-Triiodo-L-thyronine sodium salt, 1 µM ciglitazone, and 540 µM Isobutyl-1-methylxanthine) for 14 days, at which point cells were fully differentiated.

### 4.4. Rheology

Stiffness of human adipose tissue (tested within 2 h of collection) and hydrogels was measured with an HR30 Discovery Hybrid Rheometer (TA Instruments Inc., Newcastle, DE, USA), with a ‘511200.945 Plate SST ST 20 mm Smart-Swap HRx0’ geometry calibrated to manufacturer specifications to ensure accurate force and displacement measurements at a constant temperature of 20 °C. Tissue or hydrogels were placed between compression plates, aligning samples centrally to avoid off-axis loading. A pre-compression load of 0.1 N was applied to ensure proper contact between the sample and the compression plates without significantly deforming the sample, and testing was conducted at a constant linear rate of 20 µm/s, compressing until a strain of 50% was exceeded. Force and displacement data were recorded continuously and converted to stress–strain curves using the initial cross-sectional area and original height of the sample. Young’s modulus was calculated within the linear elastic region of the stress–strain curve corresponding to a strain range of 5–15%, as the slope of the linear fit within the region Ε = Δσ/Δε, where Δσ is the change in stress (Pa) and Δε is the change in strain (dimensionless). Each sample’s Young’s modulus was calculated separately, and the average Young’s modulus was reported for each sample.

### 4.5. Confocal Microscopy and Digital Analysis

Hydrogels containing differentiated adipocytes were washed twice with PBS, fixed with 4% paraformaldehyde for 1 h, 25 °C, washed twice with PBS, permeabilized with 0.5% Triton X-100 in PBS, washed twice with PBS, and blocked with 1% BSA in PBS 1 h, 25 °C, then stained with phalloidin–Alexa Fluor™ 488 (Invitrogen, Inc., Waltham, MA, USA, A12379; 1:400), HCS LipidTOX™ Red Neutral Lipid Stain (Invitrogen, Inc., Waltham, MA, USA, H34476; 1:200), and DAPI (Thermo Fisher Scientific, Inc., Waltham, MA, USA, D1306; 1 µg/mL) in PBS for 1 h, 25 °C with gentle shaking, protected from light, then washed twice with PBS and stored in PBS at 4 °C, protected from light until imaging. For lipid droplet quantification, hydrogels were placed in glass-bottom 24-well plates. Spinning-disk confocal microscopy (Nikon Corp., Tokyo, Japan) was used at 20× and 40×, and 4–8 images were taken per gel, resulting in 4–8 cells per gel. A total of 2 µm sections were taken to create a Z-stack from the bottom to the top of the adipocyte. Image analysis was performed using the Fiji image-processing package, version 2.0.0 [35]. The Z-stack was combined with a maximum-intensity projection. Cell area was calculated by manually drawing borders around cells based on the phalloidin stain. Previous methods used the Fiji water-shedding method to calculate lipid droplet size, which works well in 2D culture, but they are not accurate in 3D culture, in which lipid droplets may overlap each other, leading to underestimation of total lipid droplet area. Therefore, lipid droplet sizing was performed by manually drawing borders around each lipid droplet and calculating the area. The Fiji image-processing package was used to quantify cell shape and circularity; borders around cells were manually drawn, and circularity and area measurements were taken (Fiji ImageJ >> “Analyze” > “Set Measurements” >> in dialog box, check “Shape descriptors”, then use “Measure” command to measure the circularity. For adipogenesis, hydrogels were mounted between two coverslips. Two to three images per sample were acquired using a spinning-disk confocal microscope (Nikon Corp., Tokyo, Japan) with a 20× objective. Each image consisted of a stitched composite of nine fields. Optical sections were collected at 2 µm intervals to generate a Z-stack spanning ~50 µm. Image analysis was performed using the Fiji image-processing package [35]. For quantification of adipogenesis, a maximum-intensity projection was generated from each Z-stack. The Fiji counting tool was used to calculate the ratio of lipid droplet–positive cells with nuclei to all cells with nuclei (red: LipidTOX, blue: DAPI).

### 4.6. RNA Sequencing (RNASeq)

After 14 days of culture in adipogenic differentiation media, three 50 μL hydrogels per experimental condition were rinsed with PBS, digested in 250 U/mL Collagenase IV, 45 min, 37 °C, centrifuged, and the collagenase solution was aspirated from the cell pellet. RNA was isolated using a QIAGEN QIAshredder and RNeasy mini kit (QIAGEN, Hilden, Germany). RNA concentration was measured using a ThermoScientific NanoDrop 2000 Spectrophotometer (Waltham, MA, USA), and RNA quality was assessed using the BioAnalyzer (Agilent Inc., Santa Clara, CA, USA). Samples were stored −80 °C prior to RNASeq. For RNASeq, the SMART-Seq v4 PlUS kit (Takara Bio, Ann Arbor, MI, USA) was used to prepare libraries from 2 ng of RNA using 11 cycles of cDNA amplification and 15 cycles of library amplification per manufacturer’s protocols. Final libraries were checked for quality and quantity by Qubit hsDNA (ThermoFisher Scientific, Inc., Waltham, MA, USA) and LabChip (Perkin Elmer, Waltham, MA, USA). Samples were pooled and sequenced on the Illumina NovaSeq S4 paired-end 150 bp per manufacturer’s protocols. Data pre-processing utilized FastQC (v0.10.0) for quality control and the Tuxedo suite for alignment and quantification. Reads were aligned to the UCSC hg19 reference genome using TopHat [36,37], and gene quantification was performed with Cufflinks [38]. Ensembl-ID gene sequences were used for annotation. All subsequent analyses were performed in the R statistical programming language. Count expression data was filtered to include features with a minimum of 5 counts in at least 50% of samples. Paired-sample differential expression analysis was performed using DESeq2 (v1.8.3) [39] with default parameters. Gene set enrichment analysis was performed using the fgsea R package (v1.24.0) [40]. After differential expression analysis, genes were assigned a differential score, defined as sign (foldchange) × −log10(*p*-value), and rank-ordered. Enrichment was tested using pathway definitions from the Kyoto Encyclopedia of Genes and Genomes (KEGG) database (Release 90.0+/05-29, 19 May) [41], the Reactome Pathway Knowledgebase 2024 [42], the Molecular Signatures Database (MSigDB) Hallmark gene set collection [43], and the Gene Ontology Consortium database (2019-Apr26), using the Gene Ontogeny (GO) Biological Processes gene sets [44]. The Benjamini–Hochberg method was utilized for multiple testing corrections in all analyses. VAT and SAT samples were analyzed separately. Count data was transformed using the variance-stabilizing transformation (VST) method, and the 500 most variable genes were used in the PCA, which was conducted using DESeq2. The ggplot2 R package was used for visualization. All heatmaps were created using the pheatmap R package version 1.0.13. To improve visualization, counts for a given gene were pulled from the VST normalized count data, z-score normalized, and constrained between [−6 and 6].

### 4.7. Statistics

R version 4.5.2, R-Studio version 2025.09.2-418, and GraphPad Prism 9 were used for statistical analysis. Data normality was analyzed by the Shapiro–Wilk test. Comparisons between hydrogels for all analyses were performed using one-way or two-way ANOVA and multiple comparison analysis (Tukey). Lipid droplet size frequency data from 2.5 mM, 4.5 mM, and 7.5 mM hydrogels were pooled into VAT or SAT depots. Descriptive statistics were run to generate lipid droplet size quartiles for each depot (small: 1st quartile, medium: 2nd quartile, large: 3rd quartile, and XL: 4th quartile). Quartiles were generated for each depot separately to prevent bias due to depot differences in lipid droplet size. *p* < 0.05 was used as the threshold for statistical significance. Error bars in the Figures are the standard error of the mean.

## 5. Conclusions

Hydrogel–adipocyte culture permits tuning of matrix mechanics to manipulate adipocyte phenotype. Our data support that intermediate matrix stiffness and crosslinking maximize adipogenesis and lipid storage, while higher or lower matrix stiffness impairs these functions and instead promotes fibrotic, inflammatory, and angiogenic responses, perhaps a mechanism by which ASC remodels the matrix to restore optimal adipogenesis. These data refine our understanding of the role of mechanical properties of the ECM in regulating adipocyte function and have implications for in vitro modeling of human adipose tissue and for tissue engineering for in vivo cell-based therapy.

## Figures and Tables

**Table 1:** Subject demographics.

Subject Demographics	Male	Female
n	6	8
Age (mean, S.D.)	41 (8)	42 (11)
BMI (mean, S.D.)	42 (5)	41 (3)
HbA1c (mean, S.D.)	5.3 (0.6)	5.3 (0.2)
Hypertension (n)	2	1
Sleep apnea (n)	4	2
Hyperlipidemia (n)	3	4

## Data Availability

The data presented in this study are available on request from the corresponding author due to limitations related to human subject confidentiality.

## Supplementary Materials

The following supporting information can be downloaded at https://www.mdpi.com/article/10.3390/ijms262210865/s1.

## Abbreviations

The following abbreviations are used in this manuscript:

ASC	Adipose tissue stromal cells
DEG	Differentially expressed genes
ECM	Extracellular matrix
GO	Gene ontogeny
GSEA	Gene set enrichment analysis
LD	Lipid droplet
MMP	matrix metalloprotease
NES	Normalized enrichment score
NDM	Non-diabetic
PCA	Principal component analysis
RNASeq	RNA sequencing
SAT	Subcutaneous adipose tissue
VAT	Visceral adipose tissue
μM	Micrometer
