# Supplementary material for: A Hydrogel Culture System Regulates Human Adipocyte Function
Source: Int J Mol Sci. 2025 Nov 9;26(22):10865. doi: 10.3390/ijms262210865 (PMC12652782; doi:10.3390/ijms262210865)
Supplement: Supplementary file 1 [file ijms-26-10865-s001.zip › Kwon et al., Suppl Figure S1-FINAL, IJMS.pptx]

## Slide 1
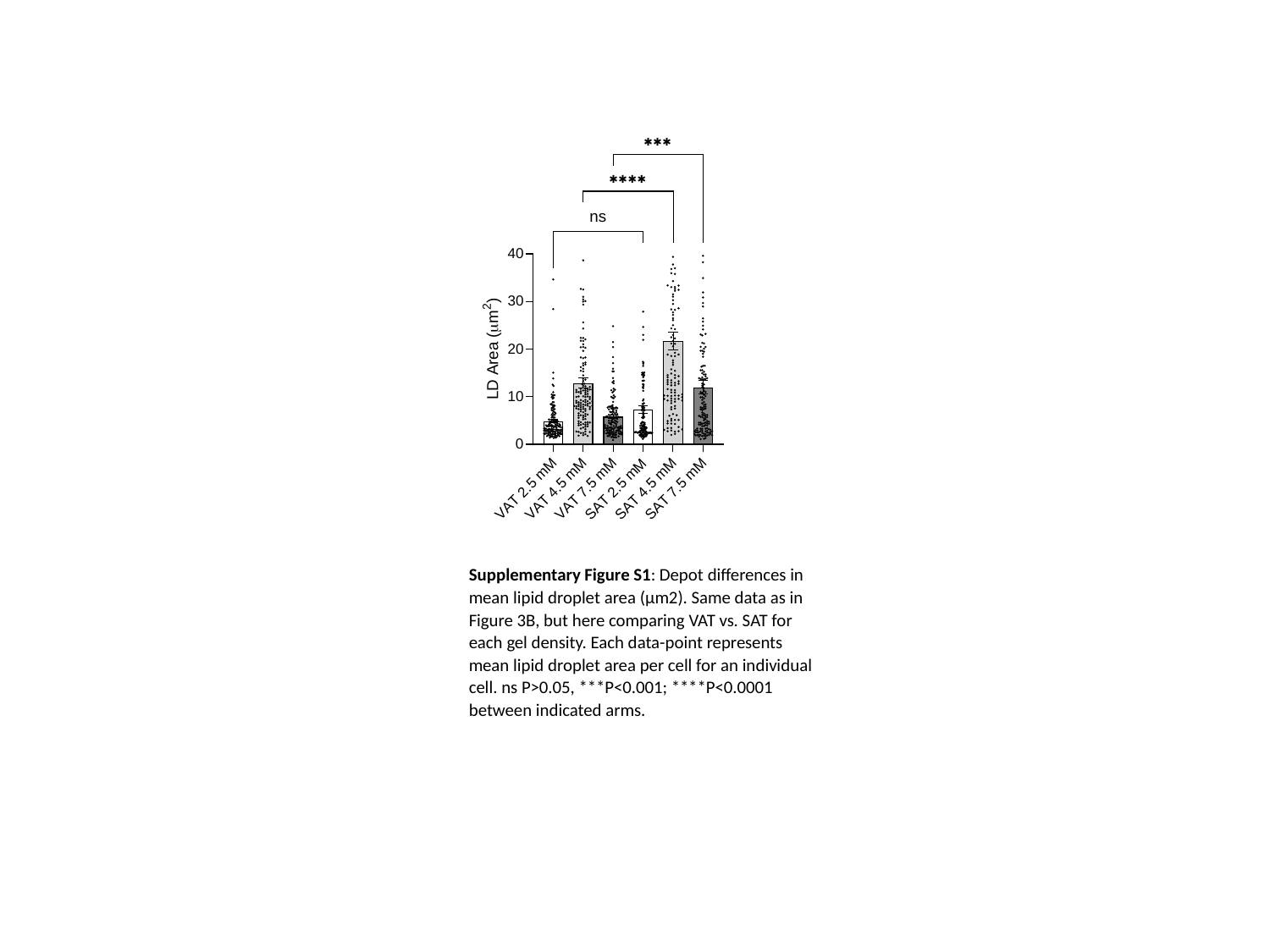

Supplementary Figure S1: Depot differences in mean lipid droplet area (μm2). Same data as in Figure 3B, but here comparing VAT vs. SAT for each gel density. Each data-point represents mean lipid droplet area per cell for an individual cell. ns P>0.05, ***P<0.001; ****P<0.0001 between indicated arms.
