# Supplementary material for: A Hydrogel Culture System Regulates Human Adipocyte Function
Source: Int J Mol Sci. 2025 Nov 9;26(22):10865. doi: 10.3390/ijms262210865 (PMC12652782; doi:10.3390/ijms262210865)
Supplement: Supplementary file 1 [file ijms-26-10865-s001.zip › Kwon et al., Suppl Figure S2-FINAL, IJMS.pptx]

## Slide 1
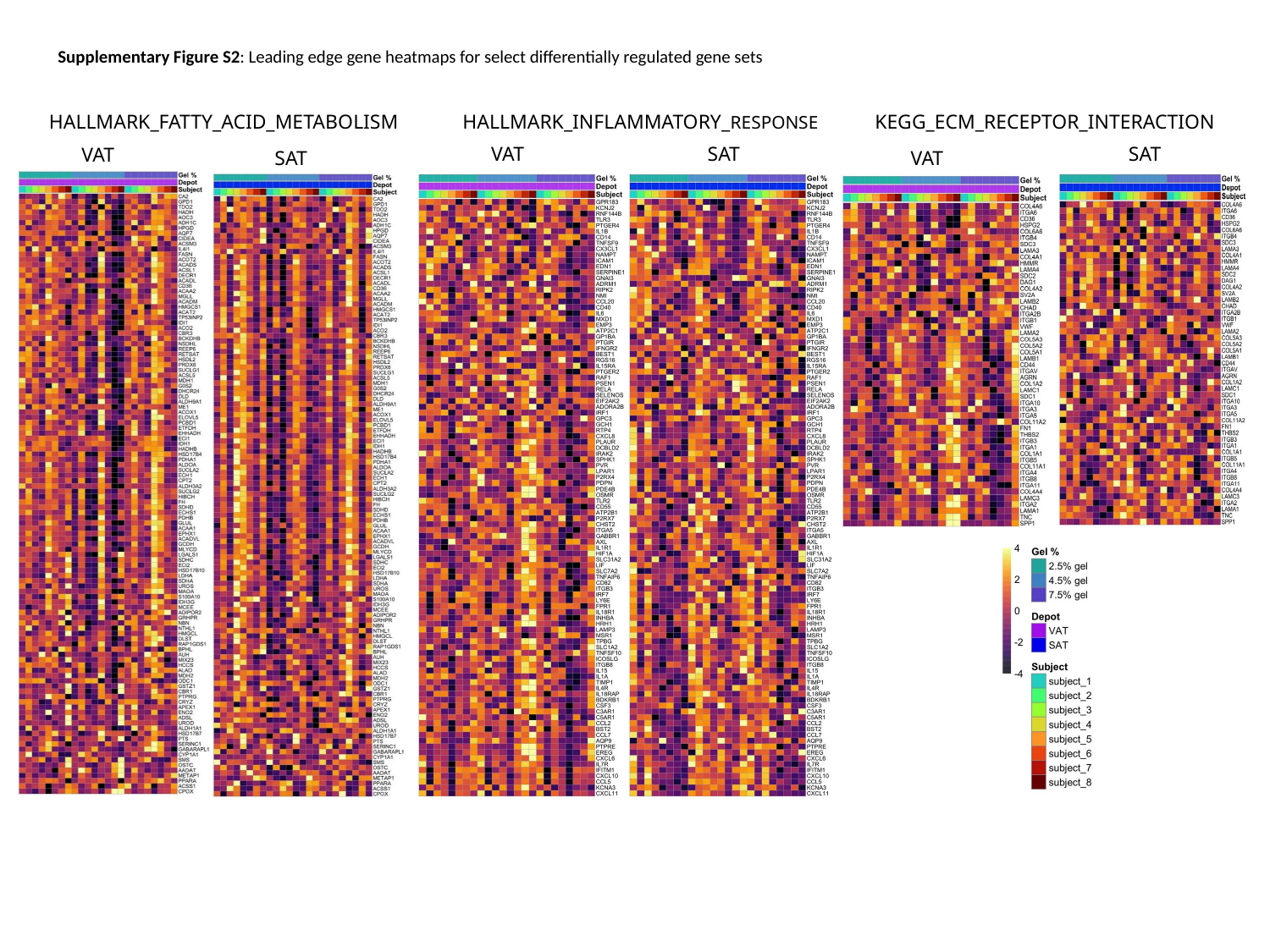

Supplementary Figure S2: Leading edge gene heatmaps for select differentially regulated gene sets
HALLMARK_FATTY_ACID_METABOLISM
HALLMARK_INFLAMMATORY_RESPONSE
KEGG_ECM_RECEPTOR_INTERACTION
VAT
SAT
SAT
VAT
SAT
VAT
